# Supplementary material for: Tempo and mode of gene expression evolution in the brain across primates
Source: eLife. 2024 Jan 26;13:e70276. doi: 10.7554/eLife.70276 (PMC10876213; doi:10.7554/eLife.70276)
Supplement: Supplementary file 3. [file elife-70276-supp3.docx]

| Species | Common Name | Complete | Fragmented | Missing |
| --- | --- | --- | --- | --- |
| *Homo sapiens* | Human | 61.3% | 8.5% | 30.2% |
| *Pan troglodytes* | Chimpanzee | 58.7% | 10.3% | 31.0% |
| *Gorilla gorilla gorilla* | Gorilla | 61.5% | 7.8% | 30.7% |
| *Lemur catta* | Ring-tailed lemur | 63.6% | 8.2% | 28.2% |
| *Ateles fusciceps* | Spider monkey | 63.2% | 8.65 | 28.2% |
| *Paio anubis* | Olive baboon | 68.5% | 7.7% | 23.8% |
| *Eulemur flavifrons* | Black lemur | 60.5% | 7.9% | 31.6% |
| *Callithrix jacchus* | Marmoset | 66.2% | 7.8% | 26.0% |
| *Macaca nemestrina* | Pig-tailed macaque | 62.2% | 8.3% | 29.5% |
| *Erythrocebus patas* | Patas monkey | 70.8% | 6.9% | 22.3% |
| *Nycticebus pygmaeus* | Pygmy slow loris | 64.0% | 7.4% | 28.6% |
| *Macaca mulatta* | Rhesus macaque | 69.8% | 7.6% | 22.6% |
| *Pithecia pithecia* | Saki | 64.7% | 6.6% | 28.7% |
| *Symphalangus syndactylus* | Siamang | 66.3% | 7.7% | 26.0% |
| *Saimiri sciureus* | Squirrel monkey | 63.6% | 7.4% | 29.0% |
| *Nycticebus coucang* | Slow loris | 58.3% | 9.9% | 31.8% |
| *Loris tardigradus* | Slender loris | 35.8% | 11.6% | 52.6% |

Table S3: BUSCO Scores against Mammalian lineage (mammalia_odb10)
